# Engineering Agatoxin, a Cystine-Knot Peptide from Spider Venom, as a Molecular Probe for In Vivo Tumor Imaging

**DOI:** 10.1371/journal.pone.0060498

**Published:** 2013-04-03

**Authors:** Sarah J. Moore, Cheuk Lun Leung, Heidi K. Norton, Jennifer R. Cochran

**Affiliations:** 1 Department of Bioengineering, Stanford University, Stanford, California, United States of America; 2 Department of Chemical Engineering, Stanford University, Stanford, California, United States of America; 3 Stanford Cancer Institute and Bio-X Program, Stanford, California, United States of America; Stanford University, United States of America

## Abstract

**Background:**

Cystine-knot miniproteins, also known as knottins, have shown great potential as molecular scaffolds for the development of targeted therapeutics and diagnostic agents. For this purpose, previous protein engineering efforts have focused on knottins based on the *Ecballium elaterium* trypsin inhibitor (EETI) from squash seeds, the Agouti-related protein (AgRP) neuropeptide from mammals, or the Kalata B1 uterotonic peptide from plants. Here, we demonstrate that Agatoxin (AgTx), an ion channel inhibitor found in spider venom, can be used as a molecular scaffold to engineer knottins that bind with high-affinity to a tumor-associated integrin receptor.

**Methodology/Principal Findings:**

We used a rational loop-grafting approach to engineer AgTx variants that bound to α_v_β_3_ integrin with affinities in the low nM range. We showed that a disulfide-constrained loop from AgRP, a structurally-related knottin, can be substituted into AgTx to confer its high affinity binding properties. In parallel, we identified amino acid mutations required for efficient in vitro folding of engineered integrin-binding AgTx variants.

Molecular imaging was used to evaluate in vivo tumor targeting and biodistribution of an engineered AgTx knottin compared to integrin-binding knottins based on AgRP and EETI.

Knottin peptides were chemically synthesized and conjugated to a near-infrared fluorescent dye. Integrin-binding AgTx, AgRP, and EETI knottins all generated high tumor imaging contrast in U87MG glioblastoma xenograft models. Interestingly, EETI-based knottins generated significantly lower non-specific kidney imaging signals compared to AgTx and AgRP-based knottins.

**Conclusions/Significance:**

In this study, we demonstrate that AgTx, a knottin from spider venom, can be engineered to bind with high affinity to a tumor-associated receptor target. This work validates AgTx as a viable molecular scaffold for protein engineering, and further demonstrates the promise of using tumor-targeting knottins as probes for in vivo molecular imaging.

## Introduction

There is a critical need for in vivo molecular imaging agents that bind specifically and with high affinity to clinical targets of interest, while displaying desirable pharmacokinetics and tissue biodistribution properties [1], [2]. For cancer, ideal molecular imaging agents are ones that exhibit robust tumor localization and rapid clearance from non-target tissues and organs [3], [4]. Such attributes translate into high imaging contrast at early time points after probe injection, and low nonspecific or background imaging signals that otherwise obscure accurate identification of malignant tissue.

**Figure 1:**
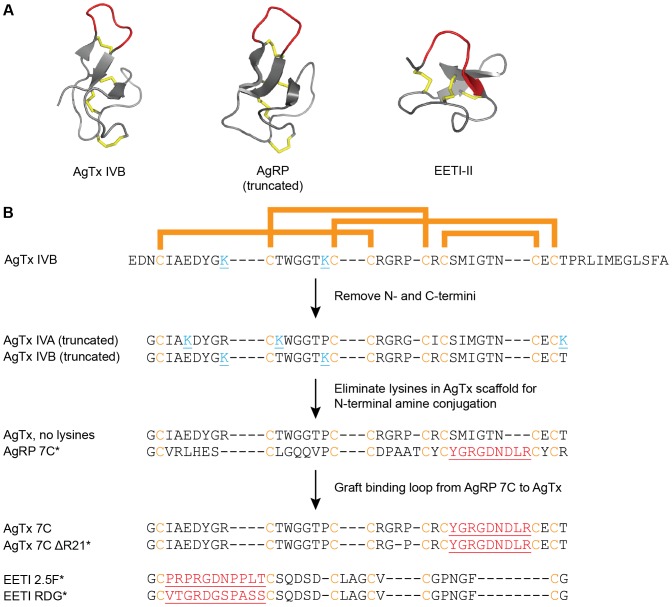
AgTx, AgRP, and EETI knottins engineered to bind tumor-associated integrins. (A) Native knottin structures. AgTx IVB (PDB 1OMB), truncated AgRP (PDB 1MR0), and EETI-II (PDB 2ETI), with disulfide bonds shown in gold, and native loops that were mutated to bind tumor-associated integrins shown in red. Structures were rendered in PyMOL. (B) Schematic of protein engineering strategy and sequences of native and engineered knottins used in this study. Conserved cysteine residues are shown in gold, and bars indicate disulfide bond connectivity. The N- and C-termini of AgTx were truncated and the sequences of isoforms IVA and IVB were combined to create a knottin scaffold with no lysine residues (cyan), allowing for site-specific conjugation of AF680 at the N-terminal amino group. The integrin-binding loop from AgRP 7C was grafted into the structurally analogous loop of this new scaffold to create AgTx 7C. Mutated loops are underlined and shown in red. * indicates knottins used for in vivo imaging. EETI RDG contains a scrambled sequence that does not bind integrins, and was used as a negative control.

Recently, cystine-knot miniproteins, known as knottins, have emerged as promising agents for non-invasive molecular imaging of tumors in living subjects [5]–[7]. Knottins share a common disulfide-bonded framework, and contain loops of variable length and composition that are constrained to a core of anti-parallel beta-strands (Fig. 1) [8]. This structure confers high thermal, chemical, and proteolytic stability [9], [10], which is desirable for in vivo biomedical applications. In addition, the small size of knottins (∼30–60 amino acids) affords rapid blood clearance and the potential for chemical synthesis, allowing facile incorporation of a variety of imaging moieties [11], [12].

Polypeptides containing cystine-knot motifs are found in myriad organisms such as plants, insects, and mammals, and carry out diverse functions including protease inhibition, ion channel blockade, and antimicrobial activity [13], [14]. Although naturally-occurring knottins have found important clinical applications [15], [16], protein engineering is playing an increasing role in creating knottins that possess novel molecular recognition properties for use as therapeutics and diagnostics [17]–[20]. The disulfide-constrained loop regions of native knottins tolerate high levels of sequence diversity (Fig. 1B), providing a robust molecular framework for engineering proteins that recognize a variety of biomedical targets. Despite the large number of natural proteins with cystine knot motifs, engineering efforts have mainly utilized three knottins as molecular scaffolds: the *Ecballium elaterium* trypsin inhibitor-II (EETI), which is found in the seeds of the squirting cucumber [21], [22]; a truncated version of the Agouti-related protein (AgRP), a neuropeptide that is involved in regulating metabolism and appetite [23], [24]; and the cyclotide Kalata B1 from the African plant *Oldenlandia*, which has uterotonic activities [25].

We previously used yeast-surface display and high-throughput library screening to identify knottin variants, based on EETI and AgRP, that possess high affinity and specificity for integrin receptors expressed on tumor cells and their neovasculature (Fig. 1) [26], [27]. Engineered integrin-binding EETI and AgRP knottins were labeled with a variety of contrast agents and used to non-invasively image tumors across multiple modalities, including positron emission tomography (PET) [28]–[34], single-photon emission computed tomography (SPECT) [35], ultrasound [36], and optical imaging [30], [31]. In these studies, engineered EETI and AgRP knottins exhibited rapid tumor localization and blood clearance via the kidneys, resulting in robust tumor contrast compared to the surrounding tissue [28]–[34]. Given the critical roles that integrins play in tumor cell survival, invasion, metastasis, and angiogenesis [37]–[40], molecular imaging agents that selectively target tumor-associated integrins have potential diagnostic applications in disease staging and management, and monitoring response to therapy [41], [42].

Spider venoms are a rich source of diverse peptides containing a cystine knot motif [43], [44]. Here, we demonstrate the first use of Agatoxin (AgTx), a venom-derived knottin [45], [46], as a molecular scaffold for protein engineering. AgRP, which is derived from mammals, is structurally homologous to AgTx, despite the fact that their sequences share only one identical residue beyond their conserved cysteines (Fig. 1). Using a rational approach, we grafted the integrin-binding loop from an engineered AgRP mutant into an AgTx scaffold, resulting in an AgTx variant that bound to tumor cells with low nanomolar affinity. Through this work, we identified scaffold mutations required for efficient in vitro folding of this engineered AgTx variant. We showed that engineered integrin-binding knottins based on AgTx, AgRP, and EETI can be conjugated to a near-infrared fluorescent dye and used for in vivo optical imaging of tumors in mice, but that EETI-based knottins had lower non-specific accumulation in the kidneys. This work validates AgTx as a molecular scaffold for protein engineering and in vivo imaging applications, expanding the repertoire of knottins that can be developed as potential therapeutic and diagnostic agents.

## Results

### Engineering a truncated AgTx scaffold that binds α_v_β_3_ integrin

EETI 2.5F is an engineered knottin that binds to α_v_β_3_, α_v_β_5_, and α_5_β_1_ integrins [26], while AgRP 7C is an engineered knottin that binds only to α_v_β_3_ integrin [27]. To develop AgTx as a scaffold for molecular engineering, we first reduced its size to minimize complexity and allow for more facile peptide synthesis. There are two primary natural isoforms, AgTx IVA and IVB, which possess 71% sequence homology [45]. These isoforms each contain 48 amino acids, including disordered N- and C-terminal regions [46]. We defined the knottin core of AgTx IVA and IVB by sequence comparison with a truncated AgRP fragment [24] that was previously used as a scaffold to engineer integrin-binding knottins [27]. As the C-terminal region of AgTx is proposed to mediate ion channel binding and inhibition [46], we removed it to mitigate potential toxicity concerns of using full-length AgTx for in vivo studies. N- and C-terminal truncations resulted in peptides containing 35 amino acids for each AgTx isoform (Fig. 1B). AgTx IVA and IVB contain 3 and 2 lysine residues, respectively, at different positions throughout the polypeptide chain. We combined the sequences of the AgTx IVA and IVB isoforms to eliminate all lysine residues, so that a molecular imaging probe could be selectively conjugated to the N-terminal amino group. We define this combined sequence as “AgTx, no lysines” (Fig. 1B). To engineer this new AgTx scaffold to bind integrins we relied on the overall structural similarity between AgTx and AgRP (Fig. 1A), and substituted the integrin-binding loop from a previously engineered knottin variant, AgRP 7C, into the analogous location within AgTx to produce AgTx 7C (Fig. 1B).

### A specific arginine deletion in the integrin-binding AgTx variant promoted efficient in vitro folding

**Figure 2:**
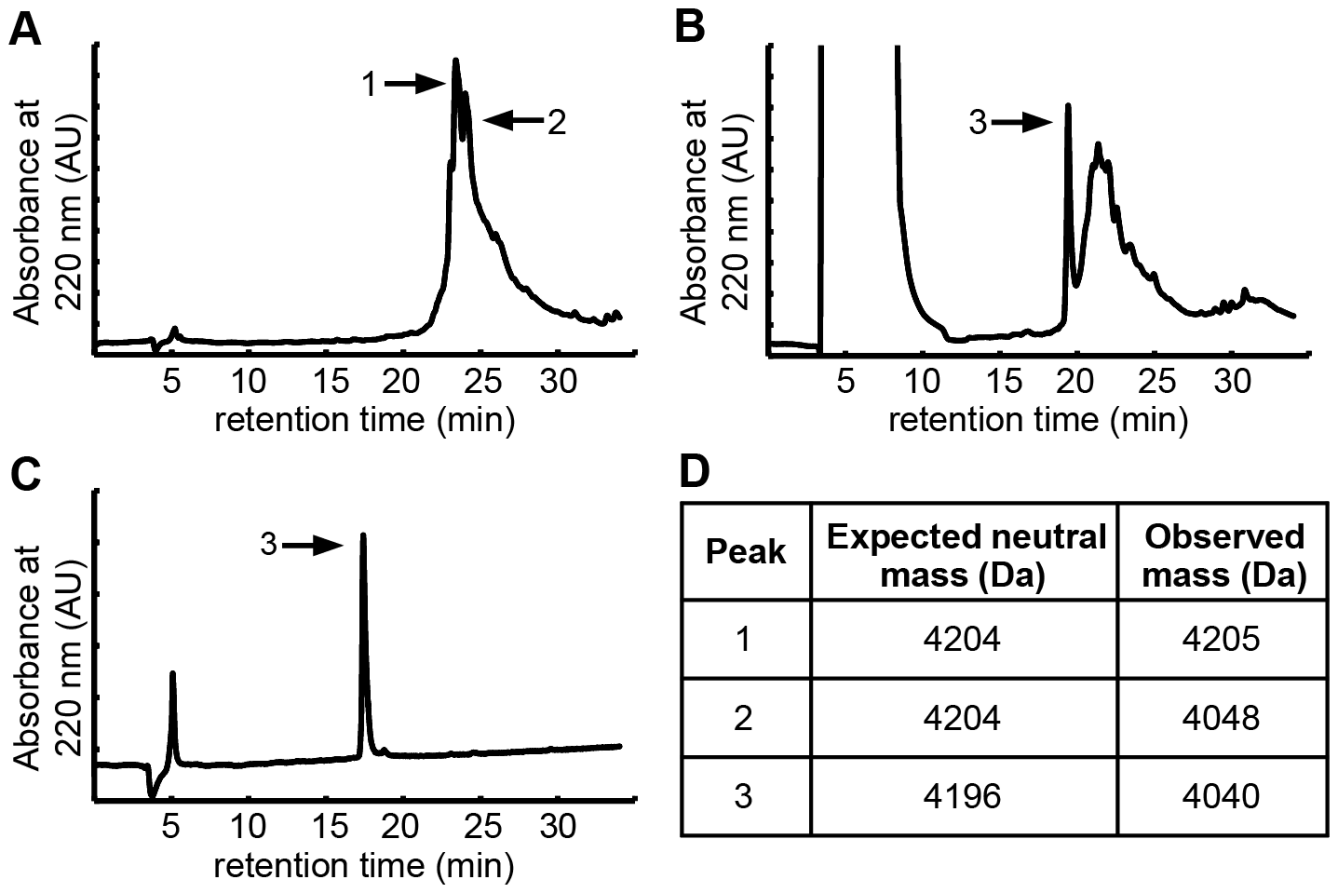
Synthesis and folding of AgTx 7C indicated a deletion product. (A–C) RP-HPLC chromatograms. (A) The crude peptide from solid-phase peptide synthesis had two major peaks, one with the expected mass and the other with a loss of 156 Da, indicating possible deletion of an arginine residue. (B) Folding of crude peptide yielded a sharp peak which was 156 Da less than the expected mass. (C) Purified, folded peptide exhibited a single, sharp peak. (D) Expected and observed masses of indicated HPLC peaks as analyzed by MALDI-TOF mass spectrometry. Note that there is an 8 Da difference between unfolded and folded AgTx 7C due to the formation of 4 disulfide bonds.

The linear precursor of the integrin-binding knottin AgTx 7C was chemically synthesized using solid-phase peptide synthesis. Crude peptide was folded in vitro using conditions previously established for disulfide bond formation with AgRP 7C [19], and the folded peptide was purified by reversed-phase high-performance liquid chromatography (RP-HPLC). Analytical-scale RP-HPLC was used to compare the crude peptide, folding reaction, and purified knottin (Fig. 2). Analysis of the folding reaction indicated the presence of a sharp elution peak (Fig. 2B), which is characteristic of folded knottin that can be separated from misfolded isomers. However, the molecular mass of this species was 156 Da less than expected, suggesting the deletion of an arginine residue (Fig. 2D). Upon further analysis, a peptide of the expected molecular mass was indeed present in the crude product of the synthesis (Fig. 2A), but efficient folding was only observed for the AgTx 7C deletion variant. This serendipitous finding prompted us to determine the identity of this AgTx 7C deletion. Enzymatic digestion and tandem mass spectrometry analysis indicated the absence of an arginine at position 21 (Fig. S1). In solid-phase peptide synthesis, coupling of an arginine residue is sometimes problematic [47], [48]. The side chain of arginine has two reactive nitrogen groups, and although one nitrogen is protected by 2,2,4,6,7-pentametyldihydrobenzofuran-5-sulfonyl (Pbf) during the synthesis, the unprotected nitrogen can form temporary intramolecular bonds, reducing coupling efficiency. The coupling efficiency is also reduced by the conformational rigidity of proline, whose unique side chain forms a covalent bond with the backbone of the growing peptide chain. Thus, the coupling of P22 followed by R21 would be expected to proceed at particularly low efficiency, potentially explaining the presence of high amounts of the AgTx 7C deletion product observed in our synthesis. To confirm these results, AgTx 7C was chemically synthesized without an arginine residue at position 21 (AgTx 7C ΔR21), resulting in a single species with the expected mass for both the purified linear precursor and the folded product (Fig. S2A, B). As further evidence that R21 was the problematic arginine residue for coupling, an AgTx variant was synthesized with an arginine deletion at position 9 (AgTx 7C ΔR9). The resulting peptide was again a mixture of two species, one with the correct mass, and one with a mass of 156 Da less than expected, presumably containing the additional R21 deletion from inefficient coupling. However, in this case we found that both AgTx peptides folded efficiently (data not shown). The cysteine adjacent to R9 forms a disulfide bond with the cysteine adjacent to P22, suggesting that the R9 deletion promotes folding through reduced steric constraints or reduced charge repulsion.

### Additional scaffold mutations promote efficient folding of AgTx 7C

To determine if other modifications to the AgTx scaffold influence folding, we synthesized additional AgTx 7C variants based on sequence variations of the AgTx isoforms. The R21 deletion is located in a region where the sequences of AgTx IVA and IVB differed. The “lysine-free” AgTx scaffold we defined included residues P22 and R24 from AgTx IVB (Fig. 1B); the corresponding residues in AgTx IVA are G22 and I24. A comparison of the AgTx IVA and AgTx IVB structures using the TM-align server [49] reveals that while overall the two structures are similar (TM-score of 0.70 and RMSD of 1.40), the region containing residues 22 and 24 has the largest variation compared to the rest of the structure. The inclusion of P22 in our combined AgTx scaffold, which directly precedes R21, might limit conformational flexibility required for efficient polypeptide folding, particularly when the 9-amino acid integrin-binding loop was incorporated into the construct. To test this hypothesis, we substituted G22 and I24 from AgTx IVA into the knottin scaffold, resulting in a variant denoted AgTx 7C P22G R24I. The mutations P22G and R24I resulted in efficient folding of AgTx 7C (Fig. S2). Moreover, only a single peptide species was produced from these syntheses, indicating that the substitution of proline with glycine at position 22 allows efficient coupling of the R21 residue. Finally, an AgTx variant incorporating P22G and R24I mutations and the ΔR21 deletion (denoted AgTx 7C ΔR21 P22G R24I) also demonstrated efficient folding (Fig S2). Folding conditions for each AgTx variant are described in the Supplemental Methods. Collectively, these results demonstrate that amino acid substitutions and deletions within the knottin scaffold can influence or promote more efficient folding.

### Engineered AgTx variants bind α_v_β_3_ integrin with high affinity

**Figure 3:**
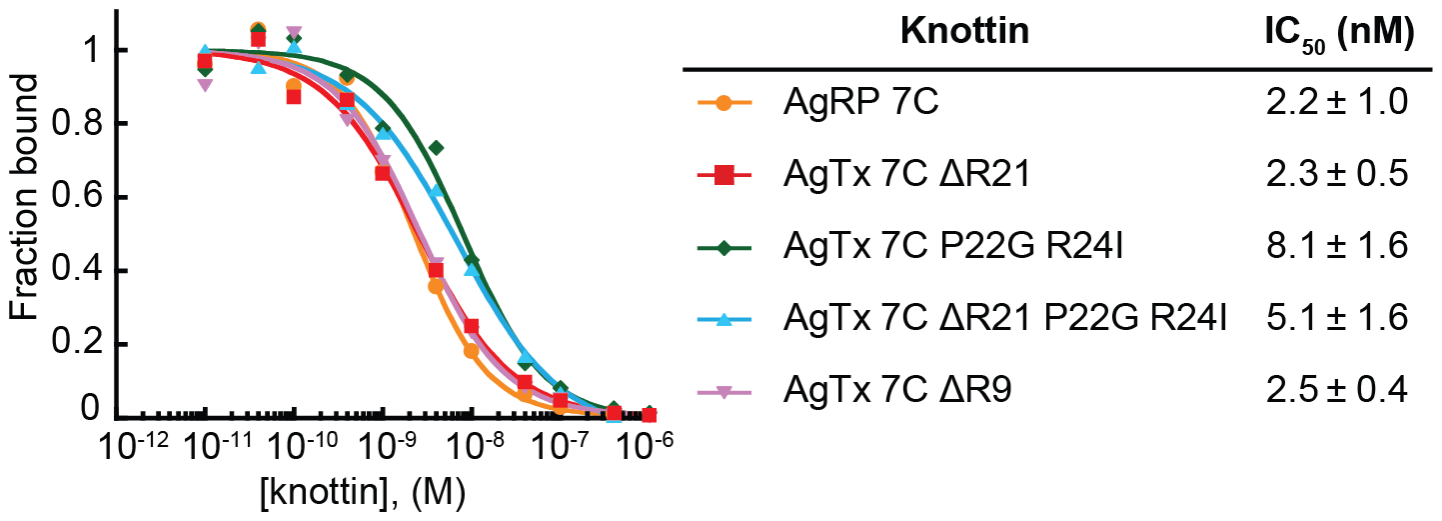
Engineered AgTx 7C variants bind to K562-α_v_β_3_ cells with similar IC_50_ values. Varying concentrations of AgRP 7C and AgTx 7C variants were incubated with FLAG-AgRP 7A and allowed to compete for binding to integrin receptors expressed on the surface of K562-α_v_β_3_ cells. Representative competition binding curves are shown, and are plotted as knottin concentration versus the fraction of FLAG-AgRP 7A bound. IC_50_ values reported as mean of three experiments ± SD.

Competition binding assays were used to measure the relative affinities of the AgTx 7C variants and AgRP 7C to K562 leukemia cells transfected to express high levels of α_v_β_3_ integrin [50]. Recombinant FLAG-AgRP 7A, a related engineered knottin that binds specifically to α_v_β_3_ integrin with high affinity [27], was used as the competitor. FLAG-AgRP 7A contains an N-terminal FLAG epitope tag (DYKDDDDK), which allows detection of cell surface binding by flow cytometry using a fluorescently labeled anti-FLAG antibody. All AgTx 7C variants showed similar relative binding affinities to K562-α_v_β_3_ cells with half-maximal inhibitory concentration (IC_50_) values in the single-digit nanomolar range (Fig. 3). AgRP 7C and AgTx 7C ΔR21 had nearly identical IC_50_ values of 2.2±1.0 nM and 2.3±0.5 nM, respectively, confirming that loop grafting of a binding epitope from AgRP to the structurally similar AgTx scaffold was a successful protein engineering strategy. As the conformation of the Arg-Gly-Asp (RGD) integrin-binding motif is critically important for mediating high-affinity interactions [51], [52], these results suggest that AgTx scaffold mutations at positions 9, 21, 22, or 24 do not significantly affect overall protein structure.

### Knottin peptides conjugated to AF680 dye retain high affinity integrin binding

**Figure 4:**
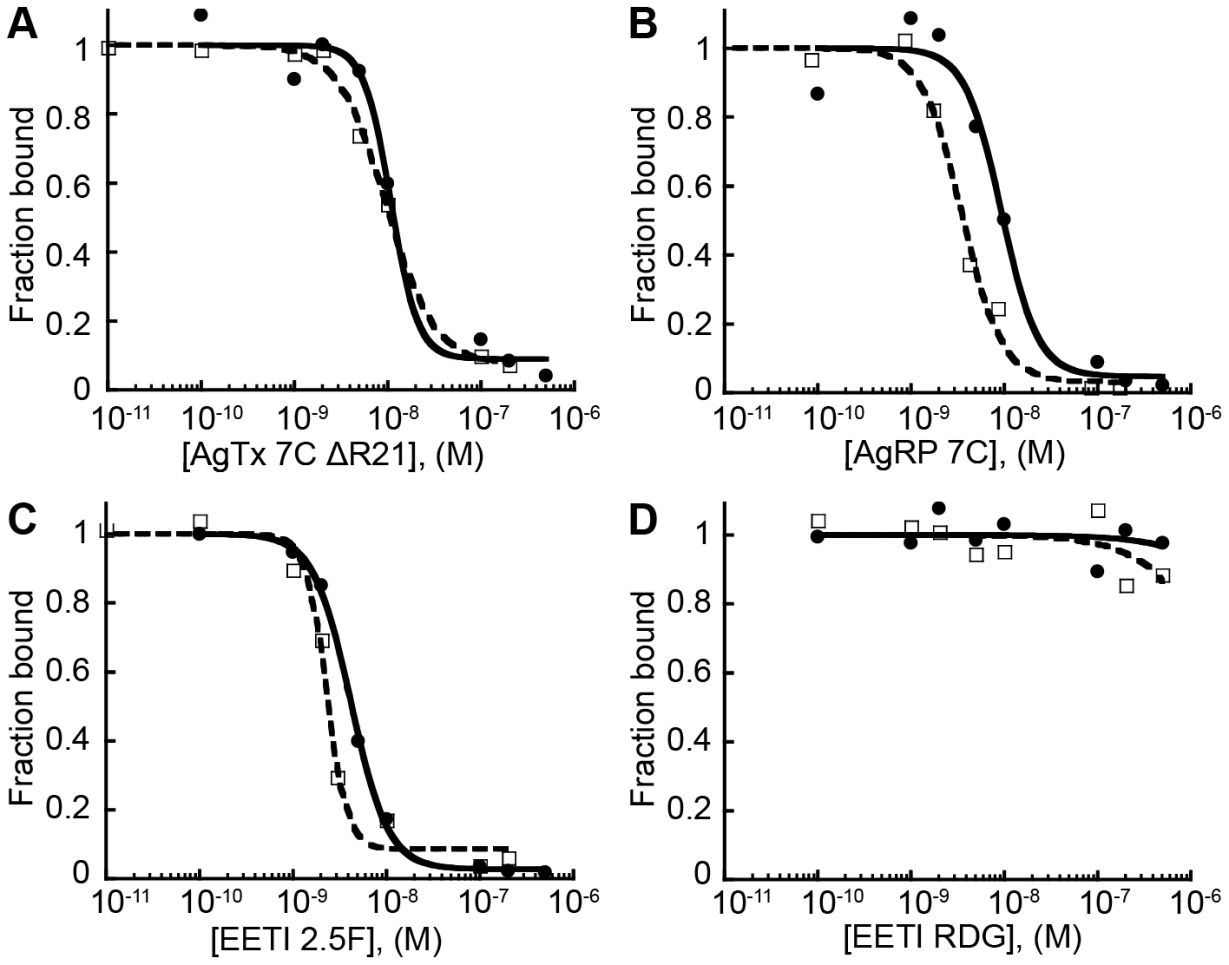
Unlabeled and AF680-labeled knottins bind U87MG cells with high affinity. Unlabeled (open squares, dashed line) and AF680-labeled (closed circles, solid line) knottins similarly compete off FLAG-AgRP 7A knottin binding to α_v_β_3_ integrins expressed on U87MG glioblastoma cells. Representative competition binding curves shown for (A) AgTx 7C ΔR21, (B) AgRP 7C, (C) EETI 2.5F, and (D) EETI RDG control. IC_50_ values reported as mean of three experiments ± SD.

For simplicity, we chose to move forward with AgTx 7C ΔR21 for in vivo molecular imaging studies. To visualize tumor targeting and tissue biodistribution, we conjugated the near-infrared dye Alexa Fluor 680 (AF680) to the N-terminal amino group of AgTx 7C ΔR21 (Fig. S3A). We also synthesized AF680-labeled versions of AgRP 7C and EETI 2.5F for comparison studies. AF680-labeled EETI RDG, which contains a scrambled integrin recognition sequence [26], [30], served as a non-binding control. AF680-labeled knottin peptides were purified by RP-HPLC and had the expected masses for the addition of one dye molecule (Fig. S3B, C). Competition binding assays were used to determine if AF680 conjugation affected integrin recognition. FLAG-AgRP 7A was used to compete for knottin binding to U87MG human glioblastoma cells (Fig. 4). Unlabeled knottins and AF680-labeled knottins had similar IC_50_ values in the low nanomolar range for each peptide tested, confirming that dye conjugation did not interfere with high-affinity integrin binding (Table 1).

**Table 1:** Relative binding affinities of unlabeled and AF680-labeled knottins on U87MG cells, reported as IC_50_ values.

	IC_50_ (nM)	
Knottin	Unlabeled	AF680 labeled
AgTx 7C ΔR21	10±1	11±3
AgRP 7C	4.0±0.4	9.2±0.2
EETI 2.5F	2.4±0.1	4.0±0.2
EETI RDG	(-)	(-)

(-)  =  no competition observed at highest concentration tested. IC_50_ values reported as mean of three experiments ± SD.

### Integrin-binding knottins exhibit high tumor contrast in murine xenograft models

**Figure 5:**
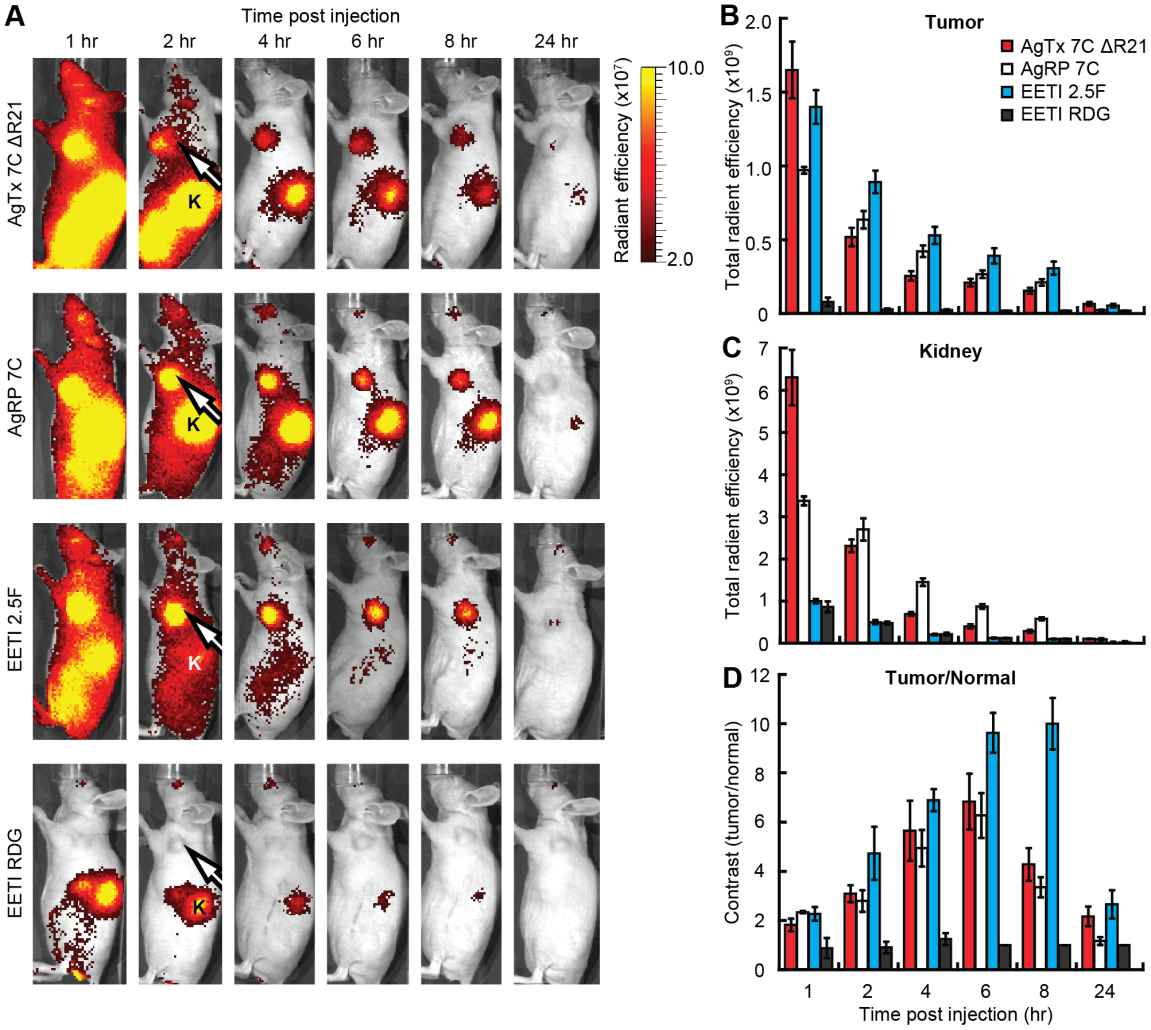
Non-invasive in vivo imaging of AF680-labeled knottins in U87MG tumor xenografts. (A) Representative whole-body fluorescent images of murine U87MG tumor xenografts injected via tail vein with 1.5 nmol AF680-labeled knottins AgTx 7C ΔR21, AgRP 7C, EETI 2.5F, and EETI RDG control. Tumors (white arrow) and kidneys (K) are indicated. Radiant efficiency [ = ] (p/s/cm^2^/sr)/(μW/cm^2^). (B–C) Quantification of imaging signals, reported as the total radiant efficiency, in the (B) tumor and (C) kidney over 24 hr. Total radiant efficiency [ = ] (p/s)/(μW/cm^2^). (D) Imaging contrast, reported as the ratio of fluorescent signals for tumor versus normal tissue. There is no statistical difference in imaging contrast between AgTx 7C ΔR21 and AgRP 7C at all time points measured (p>0.05). Error bars represent ± SE, n = 4 for all knottins.

AF680-labeled knottins were evaluated as molecular imaging probes in subcutaneous U87MG tumor xenograft models. Non-invasive optical imaging was performed over a 24 hr time period after murine tail vein injection of 1.5 nmol AF680-labeled knottin peptide (Fig. 5). Whole-body fluorescent imaging signals were prominent at 1–2 hr post-injection for AgTx 7C ΔR21, AgRP 7C, and EETI 2.5F, in contrast to the EETI RDG control, which mainly showed kidney signal due to renal clearance (Fig. 5A). For all integrin-binding knottins, tumor signals steadily decreased over the 24 hr imaging experiment (Fig. 5B). AgTx 7C ΔR21 and AgRP 7C generated high kidney imaging signals at early time points, which decreased over time (Fig. 5C). In contrast, significantly lower kidney imaging signals were observed in mice injected with EETI 2.5F and EETI RDG at all time points. EETI 2.5F generated the greatest tumor imaging contrast amongst all the knottins (Fig. 5D), as defined by the ratio of the tumor signal to the normal flank tissue of the same mouse. A comparison of AgTx 7C ΔR21 versus AgRP 7C showed similar tumor contrast throughout the imaging time course, with levels significantly higher than the EETI RDG control. Maximum tumor-to-normal tissue contrast was observed at 6–8 hr post injection after clearance of probe from non-target tissue, with values of 10.0±1.1, 6.3±0.9, and 6.8±1.1 for EETI 2.5F, AgRP 7C, and AgTx 7C ΔR21, respectively (Fig. 5D).

### EETI-based knottins generate low kidney imaging signals compared to AgRP 7C and AgTx 7C ΔR21

**Figure 6:**
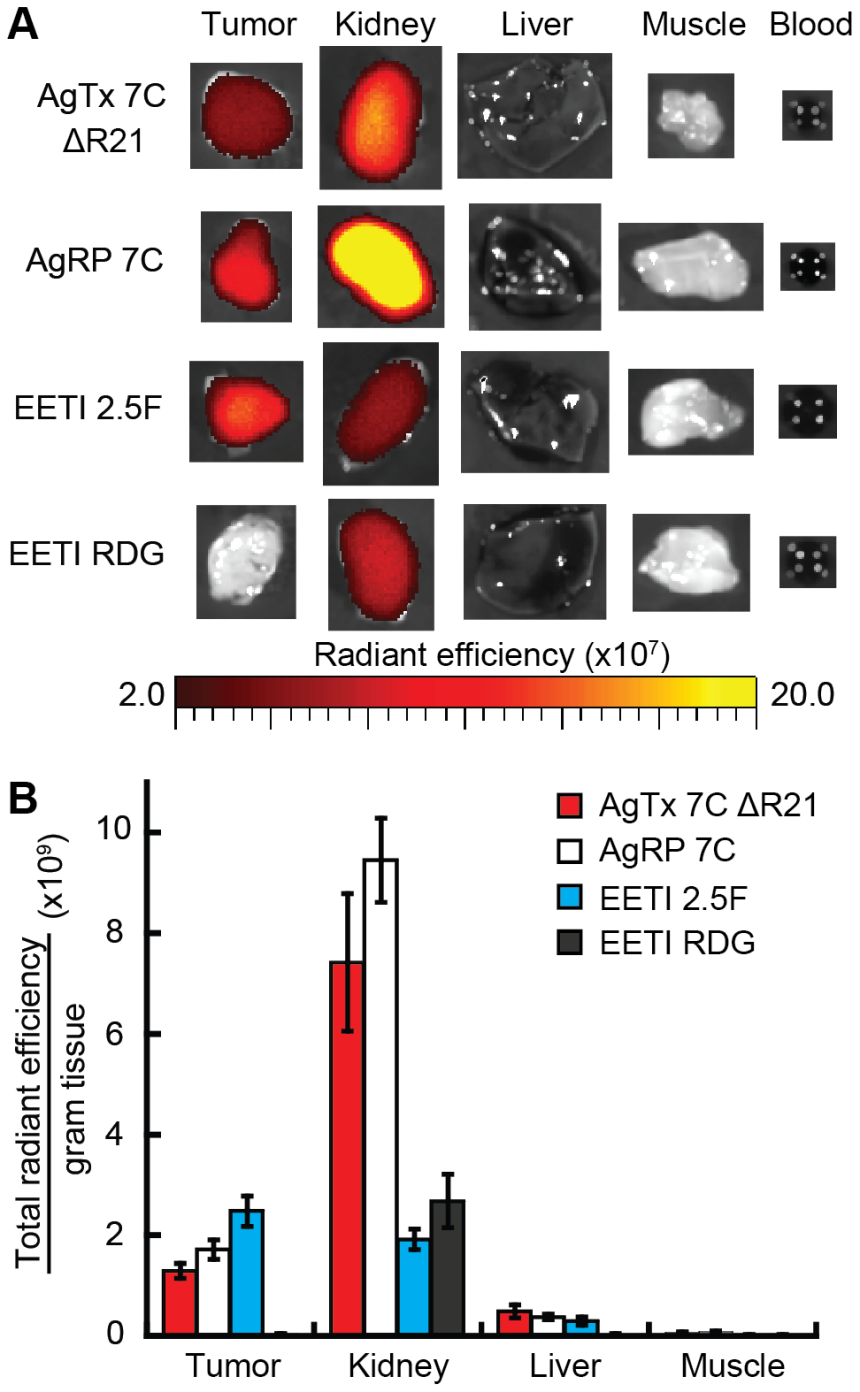
Ex vivo imaging of tissue and organs from U87MG tumor xenografts. (A) Representative ex vivo images of tumor, kidney, liver, muscle, and blood at 4 hr post injection of 1.5 nmol AF680-labeled knottins. (B) Quantification of total imaging signal per organ, normalized to organ mass, corroborates high kidney signals for AgTx 7C ΔR21 and AgRP 7C compared to EETI-based knottins. No fluorescent signal was detectable in blood samples at 4 hr post probe injection. Error bars represent ± SE. For all knottins, n = 9, except EETI RDG (n = 4).

To confirm tissue biodistribution observed with in vivo optical imaging experiments, mice were sacrificed at 4 hr and ex vivo imaging was performed on resected organs and tissue, including the tumor, kidney, liver, muscle, and blood (Fig. 6). Ex vivo imaging verified tumor-specific signals observed in mice injected with AF680-labeled AgTx 7C ΔR21, AgRP 7C, and EETI 2.5F compared to the EETI RDG control. In agreement with non-invasive in vivo optical imaging experiments, EETI 2.5F and EETI RDG generated low kidney signals at 4 hr post injection compared to AgTx 7C ΔR21 and AgRP 7C. In all animals, fluorescent signal was negligible in the muscle, blood, and liver, confirming efficient clearance from these organs and tissue.

## Discussion

Previous protein engineering studies have focused on the development and application of knottin scaffolds based on EETI, AgRP, and Kalata B1 [5]–[7], [17], [18], [28]. Our current work expands the examples of knottins validated as molecular scaffolds to include AgTx. We first truncated the N- and C-termini of AgTx IVB to simplify the scaffold and abolish its native function. Based on structural similarities between AgRP and AgTx knottins, we next grafted a disulfide-bonded integrin-binding loop from an engineered AgRP variant into AgTx, and showed that high-affinity integrin binding was conferred with this new construct. A similar approach could potentially be used to incorporate disulfide-bonded cyclic peptides, such as those identified from phage display libraries [53], into knottin scaffolds.

Through this work, we identified an arginine deletion (ΔR21) that was required for efficient folding of the integrin-binding variant AgTx 7C. In contrast, native AgTx did not require this ΔR21 mutation for efficient folding (data not shown). The engineered integrin-binding loop we introduced into AgTx 7C is 9 amino acids in length, while the corresponding loop in native AgTx consists of 6 amino acids. This longer loop, together with the inclusion of P22 which directly precedes the R21 mutation, may have disrupted an optimal balance of flexibility, size, and charge of amino acid side chains critical to folding amongst these isoforms. As a result of these changes, mutations immediately preceding the engineered integrin-binding loop were required for efficient folding. The AgTx mutations ΔR21 and/or P22G and R24I could potentially restore the thermodynamic favorability of knottin folding by removing steric clashes, improving backbone flexibility, or improving interaction of the beta strands in the knottin core. All of these AgTx 7C variants had similar relative binding affinities (Fig. 3), demonstrating that the scaffold modifications did not significantly affect the conformation of the engineered integrin-binding loop. AgRP 7C demonstrated efficient folding without the requirement for additional scaffold mutations. In contrast to the AgTx variants, AgRP has two Cys-Tyr-Cys motifs that flank the engineered integrin-binding loop. These Tyr residues have been proposed to participate in interactions that help mediate disulfide bond formation in native AgRP [54]. In addition, the Arg21 and Pro22 amino acid residues that were problematic for AgTx folding are Ala and Thr, respectively, in AgRP, which likely imparts conformational flexibility that better accommodates the engineered integrin-binding loop.

A myriad of small molecules and peptides containing an RGD integrin-binding motif have been developed, and several have advanced to human trials for diagnostic applications [55], [56]. We showed that engineered integrin-binding knottins generate significantly higher levels of tumor contrast in subcutaneous U87MG xenograft models compared to imaging probes based on c(RGDfK) and c(RGDyK), two well-characterized cyclic pentapeptides that bind to α_v_β_3_ and α_v_β_5_ integrins. Maximum tumor-to-normal tissue contrast ratios for AF680-c(RGDfK) and AF680-c(RGDyK) were 3.2±0.5 and 2.8±0.3, respectively (Fig. S4), compared to imaging contrast ratios of 6-10 for AF680-labeled integrin-binding knottins (Fig. 5D). In addition to the potential for improved tumor contrast, molecular imaging probes can be directly conjugated to the knottin N-terminus without disrupting receptor binding interactions. The N-terminus of AgTx 7C ΔR21 is positioned on the opposite side of the knottin relative to the integrin-binding loop (Fig. 1). As such, conjugation of a fluorescent dye molecule to the N-terminus of AgTx 7C ΔR21 did not interfere with high affinity binding to cell surface integrin receptors. Similar results were obtained with imaging probe conjugation to the N-terminus of AgRP 7C and EETI 2.5F. In comparison, small molecules and disulfide-constrained cyclic peptides present binding epitopes within a more limited framework that can sometimes be disrupted upon imaging probe conjugation [53], [55].

Another goal of our study was to compare the in vivo biodistribution profiles of engineered knottins derived from different organisms, including spiders, mammals, and plants. AF680-labeled AgTx 7C ΔR21, AgRP 7C, and EETI 2.5F all exhibited high tumor contrast in optical imaging experiments. The high tumor-to-normal imaging signals observed at early time points after knottin administration are consistent with theoretical and experimental studies examining the relationship between optimal molecular size and affinity needed for effective tumor targeting [57]–[60]. In particular, while small proteins accumulate rapidly in tumors, they must bind their targets with high affinity to be retained. Together, these results support further development of low molecular weight, high affinity tumor-targeting agents for molecular imaging applications.

Integrin-binding knottins based on the EETI scaffold exhibited remarkably low kidney signals, either due to more rapid clearance or reduced non-specific kidney accumulation compared to AgTx 7C ΔR21 and AgRP 7C. These results are consistent with our previous imaging studies using radiolabeled EETI and AgRP proteins [28]–[34]. In comparison, molecular imaging probes based on antibodies, antibody fragments, and other “alternative scaffolds” including affibodies, DARPins, and fibronectin domains, all exhibit high imaging signals in the liver and/or kidneys [57], [61]–[64], which raises toxicity concerns and challenges for imaging the abdominal and thoracic regions. Such high kidney signals have been attributed to metabolites that accumulate in the kidneys through mechanisms such as renal tubular reabsorption [65], [66]. We previously showed that AgRP 7C exhibits higher levels of metabolic breakdown in the liver, kidneys, and tumor compared to EETI 2.5F, although both probes were remarkably stable in serum and blood and were excreted intact in the urine [29], [30].

The amino acid sequences of the knottin peptides could also play a role in their tissue biodistribution properties. In addition to having different scaffold sequences, the engineered loops of EETI 2.5F (PRPRGDNPPLT) and EETI RDG (VTGRDGSPASS) are markedly different from that of AgRP/AgTx 7C (YGRGDNDLR). In agreement with this hypothesis, our colleagues recently reported that highly charged residues, particularly arginine and glutamic acid, of *Momordica cochinchinensis* trypsin inhibitor-II knottins contribute greatly to non-specific kidney retention [67]. In addition, an ^111^In-labeled version of one of our alternative engineered integrin-binding AgRP knottins (AgRP 6E, engineered loop sequence: VERGDGNRR) had an approximately 50% reduction in kidney signal compared to ^111^In-labeled AgRP 7C [35], demonstrating the influence of the engineered loop on tissue biodistribution. Efforts to reduce undesirable non-target tissue accumulation have included: 1) introducing mutations that increase hydrophilicity and remove charged groups, 2) covalently attaching polymers such as polyethylene glycol, and 3) co-administration of the probe with charged amino acids or agents that block specific transporter receptors or alter overall kidney physiology [65], [66], [68]. The complexity of potential mechanisms for probe or metabolite retention in the kidneys highlights the need for polypeptides that do not require extensive efforts to block renal accumulation. Thus, in future studies it will be interesting to further explore the relationship between amino acid sequence, metabolic stability, and in vivo biodistribution properties of engineered knottins.

The methods we report for chemical synthesis, folding, dye conjugation, and purification of knottins as molecular imaging probes are compatible with production of clinical grade material, although yield has not yet been evaluated in a large-scale (>1 gram) production process. The homogeneity of the final product, with one fluorophore attached to the N-terminus of each knottin peptide, is desirable for clinical translation. Knottins are proposed to be non-immunogenic due to their high stability, which is thought to preclude presentation of peptide fragments to molecules that mediate immune function [69]. In addition, smaller quantities of knottin are used for in vivo molecular imaging compared to therapeutic dosage levels; however, immunogenicity and toxicity will need to be evaluated for each compound intended for human clinical trials.

In summary, we demonstrate that AgTx, an ion channel inhibitor found in spider venom, can be used as a pliable framework for constructing peptides that bind with high-affinity to a clinically-relevant integrin receptor. This work expands the repertoire of knottins that have been validated as molecular scaffolds for protein engineering, and provides insights for the further development of engineered knottins as in vivo molecular imaging agents.

## Materials and Methods

### Ethics statement

All animal procedures were in compliance with Protocol 22942 approved by the Stanford University Administrative Panels on Laboratory Animal Care. All procedures were conducted while animals were under general anesthesia with isofluorane, and all efforts were made to minimize suffering.

### Materials, reagents, and cell lines

Integrin binding buffer (IBB) was composed of 20 mM Tris (pH 7.5) with 1 mM MgCl_2_, 1 mM MnCl_2_, 2 mM CaCl_2_, 100 mM NaCl, and 1 mg/mL bovine serum albumin (BSA). BPBS buffer was composed of phosphate-buffered saline (PBS) and 1 mg/mL BSA. 9-fluorenylmethyloxycarbonyl (Fmoc)-protected amino acids were purchased from Novabiochem/EMD Chemicals Inc. or CS Bio. The cyclic pentapeptides c(RGDfK) and c(RGDyK) were purchased from Peptides International. Human U87MG glioblastoma cells (ATCC) were cultured in DMEM (Gibco) supplemented with 10% fetal bovine serum (FBS) and 1% penicillin-streptomycin (Gibco). K562 cells transfected with α_v_β_3_ integrins (courtesy of S. Blystone) were cultured in IMDM (Gibco) supplemented with 1 mg/ml active geneticin (Gibco), 10% FBS, and 1% penicillin-streptomycin [50].

### Peptide synthesis, folding, and purification

Knottin peptides were prepared as previously described in detail [19]. Briefly, linear precursor peptides were synthesized on a CS Bio CS336 instrument using Fmoc-based solid-phase peptide synthesis. After side-chain deprotection and resin cleavage, peptides were folded by promoting disulfide bond formation in oxidation buffers optimized for each peptide (Text S1). Folded knottins were purified by preparative-scale RP-HPLC using a Varian Prostar instrument and Vydac C_18_ columns, where each folded peptide eluted as a sharp peak with an altered retention time from unfolded or misfolded precursors. Linear gradients of 90% acetonitrile in water containing 0.1% (v/v) trifluoroacetic acid were used for all peptide purifications, which were monitored at absorbances of 220 nm and 280 nm. Peptide purity was analyzed by analytical-scale RP-HPLC using a Vydac C_18_ column. Molecular masses were determined by matrix-assisted laser desorption/ionization time-of-flight mass spectrometry (MALDI-TOF-MS; Stanford Protein and Nucleic Acid Facility) or electrospray ionization mass spectrometry (ESI-MS; Stanford Vincent Coates Foundation Mass Spectrometry Laboratory). Following purification, folded knottins were lyophilized and stored at room temperature until used. Purified knottins were dissolved in PBS, and concentrations were determined by amino acid analysis (UC Davis Proteomics Core Facility).

### AF680 dye conjugation

Pure, folded knottin (2 mg/ml) was incubated for 1 hr at room temperature and then at 4 °C overnight (with stirring) with Alexa Fluor 680 carboxylic acid, succinimidyl ester (Invitrogen) in a 0.1 M sodium bicarbonate solution, pH 8.0 at a 5∶1 dye/peptide molar ratio (Fig. S3A). The resulting dye-conjugated knottins were purified by RP-HPLC (Fig. S3C). Masses were confirmed by MALDI-TOF mass spectrometry (Fig. S3B). AF680-labeled c(RGDfK) and c(RGDyK) were prepared in a similar manner. AF680-labeled compounds were lyophilized and resuspended in PBS, and concentrations were determined using UV-Vis spectroscopy, measuring dye absorption at 679 nm (ε = 184,000 cm^−1^M^−1^). Alternatively, AF680-labeled knottins were purified by extensive buffer exchange with PBS using a centrifugal filter unit with a 3 kDa molecular weight cutoff (Amicon). In vivo imaging results were consistent between knottins purified through these two methods. AF680-labeled compounds, at a concentration of 15 µM in PBS, were passed through a 0.22 µm filter for animal experiments.

### Cell binding assays

Competition binding assays were performed on K562 leukemia cells transfected to express high levels of α_v_β_3_ integrins [50]. Varying concentrations of AgRP 7C and AgTx variants were incubated with 5×10^4^ K562-α_v_β_3_ cells in IBB for 4 hr at 4 °C, along with a constant concentration (0.5 nM) of recombinantly expressed FLAG-AgRP 7A knottin as a competitor. FLAG-AgRP 7A binds with high affinity to α_v_β_3_ integrin and contains an N-terminal epitope tag (DYKDDDDK), allowing cell surface binding to be detecting using an anti-FLAG antibody [27]. Care was taken to allow adequate time for equilibrium binding and to avoid ligand depleting conditions. After incubation with knottins, cells were washed with BPBS and resuspended with a 1∶100 dilution of R-PE-conjugated anti-FLAG antibody (Prozyme) for 30 min on ice. Cells were washed with BPBS and analyzed by flow cytometry on a FACSCalibur instrument (BD Biosciences), and data was quantified using FlowJo software (TreeStar). IC_50_ values were determined by nonlinear regression analysis using KaleidaGraph (Synergy Software). Similar competition binding assays were performed on U87MG glioblastoma cells, which express high levels of α_v_β_3_ integrin receptors [70]. EETI 2.5F, AgRP 7C, AgTx 7C ΔR21, or EETI RDG, unlabeled or site-specifically labeled with one molecule of AF680 dye, were used for binding experiments. Varying concentrations of knottins were incubated with 5×10^4^ U87MG cells in IBB for 4 hr at 4 °C with a constant concentration (5 nM) of FLAG-AgRP 7A as a competitor. Cell washes, secondary antibody incubation, and data collection and analysis were performed as for the K562-α_v_β_3_ binding assays. IC_50_ values are reported as the mean and standard deviation of at least three separate experiments.

### Mouse handling and generation of tumor xenograft models

Animal procedures were carried out on 4-week old female nude mice (Charles River Laboratory). 5×10^6^ U87MG cells, suspended in 50 µl PBS along with 50 µl of Matrigel Basement Membrane Matrix (BD Biosciences, cat# 354234), were injected subcutaneously into the left shoulder of mice to generate human tumor xenografts. Mice were imaged when tumors reached 5–10 mm in diameter.

### In vivo and ex vivo optical imaging

Mice bearing U87MG tumor xenografts were anesthetized with isofluorane and injected via tail vein with 1.5 nmol AF680-labeled knottins, c(RGDfK), or c(RGDyK) in 100 µl of PBS. Whole-body in vivo fluorescence imaging was performed at the indicated times after probe injection using an IVIS 200 system (Caliper Life Sciences). The near-infrared fluorophore AF680 was excited at 615–665 nm and emission was analyzed at 695–770 nm. Background autofluorescence emission signal was also collected by exciting at 580–610 nm and analyzing at 695–770 nm. In each imaging set, a mouse injected with PBS alone (no knottin) was included to allow measurement of background signals for data processing. For ex vivo imaging, mice were sacrificed, and organs were excised and imaged using the same excitation and emission wavelengths as for in vivo imaging. Excised organs were weighed to determine the total fluorescent signal flux/gram of tissue.

### Imaging quantitation

All optical imaging quantification was performed using Living Image software (Caliper Life Sciences). In vivo signal was calculated as [Emission_615–665 nm Excitation_]–k*[Emission_580–610 nm Excitation_], where the constant k was determined such that a background region of interest (ROI) would have no signal. This background ROI was drawn around the tumor of the control mouse in each experiment that received a PBS injection with no knottin. Contrast was calculated as total radiant efficiency (units of [photons/sec/cm^2^/steradian]/[µW/cm^2^]) for tumor tissue, divided by total radiant efficiency for an ROI on normal flank tissue on the same mouse. For ex vivo quantification, total radiant efficiency was measured for the entire organ, and normalized by the mass of the organ to determine flux/g of tissue. Two-tailed Student's t-tests were used to evaluate and assign statistical significance between data sets.

## Supporting Information

Figure S1 **Enzymatic digestion and tandem mass spectrometry analysis of folded AgTx 7C reveals an arginine deletion at position 21.** The modified AgTx 7C knottin (observed mass = 4040 Da) was reduced with dithiothreitol and alkylated with iodoacetamide. (A) Comparison of MALDI-TOF-MS of a chymotryptic digest to *in silico* chymotrypsin digestion using ExPASy PeptideMass revealed that the mass discrepancy is located in the sequence GGTPCCRGRPCRCY (position 13–26). (B) Comparison of tryptic digest to a Mascot search revealed that Arg19 is present and Arg24 is likely present due to the existence of fragment 25–34, indicating cleavage by trypsin after residue 24. This data suggests that the missing Arg is located at residue 21. (C) MS/MS analysis of the 1600 Da chymotryptic peptide further supports the sequence GGTPCCRG_PCRCY, with Arg21 as the most likely deletion, by the observation of y_3_, y_5_, y_6_, y_7_, and y_8_ ions.
(TIF)

Figure S2 **Modifications to the AgTx scaffold promote in vitro folding of integrin-binding variants.** Analytical-scale RP-HPLC traces of linear, crude peptide (left), folding reaction (center), and purified, folded peptide (right) for AgTx 7C variants. Yield of purified, folded AgTx 7C was too low for further analysis. AgTx 7C P22G R24I and AgTx 7C ΔR21 P22G R24I were efficiently separated from misfolded isomers when folded from purified, linear precursor peptide, but not when folded from unpurified, crude peptide under the conditions tested. Thus, for these variants, crude linear peptide was first purified by preparatory-scale RP-HPLC using a Vydac C_18_ column before folding. In contrast, purification of the AgTx 7C linear precursor prior to folding still resulted in very low folding efficiency. (B) Masses of folded, purified knottins were determined by ESI-MS or MALDI-TOF-MS.
(TIF)

Figure S3 **AF680 conjugation and characterization.** (A) The near infrared dye AF680 was site-specifically conjugated to knottins at their N-terminal amino group using succinimidyl ester chemistry. (B) Folded, purified knottins and AF680-labeled knottins were analyzed by mass spectrometry. Expected error in these measurements is 0.1%. (C) Analysis of purified AF680-labeled knottins by analytical-scale RP-HPLC. Purity was determined to be greater than 95%. Blue traces: absorbance at 220 nm by amide bonds, red traces: absorbance at 675 nm by AF680 fluorophore.
(TIF)

Figure S4 **Non-invasive in vivo imaging with AF680-labeled cyclic RGD peptidomimetics.** (A) Mice bearing U87MG tumor xenografts were injected with 1.5 nmol AF680-c(RGDfK) or AF680-c(RGDyK), which exhibited high tumor uptake but slow clearance from non-target tissues. Tumors (white arrow) and kidneys (K) are indicated. (B) Maximum tumor-to-normal tissue contrast ratios of 3.2±0.5 and 2.8±0.3 were measured for AF680-c(RGDfK) and AF680-c(RGDyK), respectively. Error bars represent ± SE, n = 3.
(TIF)

Text S1 **Supplemental materials and methods.**
(DOCX)

## References

[1] Reynolds F, Kelly KA (2011) Techniques for molecular imaging probe design. Mol Imaging 10: 407–419. 22201532 PMC3224676

[2] Chen K, Chen X (2010) Design and development of molecular imaging probes. Curr Top Med Chem 10: 1227–1236. 20388106 10.2174/156802610791384225 PMC3632640

[3] Friedman M, Stahl S (2009) Engineered affinity proteins for tumour-targeting applications. Biotechnol Appl Biochem 53: 1–29. 19341363 10.1042/BA20080287

[4] Batra SK, Jain M, Wittel UA, Chauhan SC, Colcher D (2002) Pharmacokinetics and biodistribution of genetically engineered antibodies. Curr Opin Biotechnol 13: 603–608. 12482521 10.1016/s0958-1669(02)00352-x

[5] Daly NL, Craik DJ (2011) Bioactive cystine knot proteins. Curr Opin Chem Biol 15: 362–368. 21362584 10.1016/j.cbpa.2011.02.008

[6] Kolmar H (2011) Natural and engineered cystine knot miniproteins for diagnostic and therapeutic applications. Curr Pharm Des 17: 4329–4336. 22204431 10.2174/138161211798999465

[7] Moore SJ, Leung CL, Cochran JR (2011) Knottins: Disulfide-bonded therapeutic and diagnostic peptides. Drug Discovery Today: Technologies 9: e3–e11. 10.1016/j.ddtec.2011.07.003 24064239

[8] Pallaghy PK, Nielsen KJ, Craik DJ, Norton RS (1994) A common structural motif incorporating a cystine knot and a triple-stranded beta-sheet in toxic and inhibitory polypeptides. Protein Sci 3: 1833–1839. 7849598 10.1002/pro.5560031022 PMC2142598

[9] Colgrave ML, Craik DJ (2004) Thermal, chemical, and enzymatic stability of the cyclotide kalata B1: the importance of the cyclic cystine knot. Biochemistry 43: 5965–5975. 15147180 10.1021/bi049711q

[10] Werle M, Schmitz T, Huang HL, Wentzel A, Kolmar H, et al (2006) The potential of cystine-knot microproteins as novel pharmacophoric scaffolds in oral peptide drug delivery. J Drug Target 14: 137–146. 16753827 10.1080/10611860600648254

[11] Miao Z, Levi J, Cheng Z (2010) Protein scaffold-based molecular probes for cancer molecular imaging. Amino Acids epub Feb 12. 10.1007/s00726-010-0503-9 PMC2914822 20174842

[12] Reinwarth M, Nasu D, Kolmar H, Avrutina O (2012) Chemical Synthesis, Backbone Cyclization and Oxidative Folding of Cystine-knot Peptides; Promising Scaffolds for Applications in Drug Design. Molecules 17: 12533–12552. 23095896 10.3390/molecules171112533 PMC6268209

[13] Zhu S, Darbon H, Dyason K, Verdonck F, Tytgat J (2003) Evolutionary origin of inhibitor cystine knot peptides. FASEB J 17: 1765–1767. 12958203 10.1096/fj.02-1044fje

[14] Gracy J, Le-Nguyen D, Gelly JC, Kaas Q, Heitz A, et al (2008) KNOTTIN: the knottin or inhibitor cystine knot scaffold in 2007. Nucleic Acids Res 36: D314–319. 18025039 10.1093/nar/gkm939 PMC2238874

[15] Williams JA, Day M, Heavner JE (2008) Ziconotide: an update and review. Expert Opin Pharmacother 9: 1575–1583. 18518786 10.1517/14656566.9.9.1575

[16] Veiseh M, Gabikian P, Bahrami SB, Veiseh O, Zhang M, et al (2007) Tumor paint: a chlorotoxin:Cy5.5 bioconjugate for intraoperative visualization of cancer foci. Cancer Res 67: 6882–6888. 17638899 10.1158/0008-5472.CAN-06-3948

[17] Craik DJ, Swedberg JE, Mylne JS, Cemazar M (2012) Cyclotides as a basis for drug design. Expert Opin Drug Discov 7: 179–194. 22468950 10.1517/17460441.2012.661554

[18] Kolmar H (2010) Engineered cystine-knot miniproteins for diagnostic applications. Expert Rev Mol Diagn 10: 361–368. 20370592 10.1586/erm.10.15

[19] Moore SJ, Cochran JR (2012) Engineering knottins as novel binding agents. Methods in Enzymology 503: 223–251. 22230571 10.1016/B978-0-12-396962-0.00009-4

[20] Getz JA, Rice JJ, Daugherty PS (2011) Protease-resistant peptide ligands from a knottin scaffold library. ACS Chem Biol 6: 837–844. 21615106 10.1021/cb200039s PMC3158827

[21] Favel A, Mattras H, Coletti-Previero MA, Zwilling R, Robinson EA, et al (1989) Protease inhibitors from *Ecballium elaterium* seeds. Int J Pept Protein Res 33: 202–208. 2654042 10.1111/j.1399-3011.1989.tb00210.x

[22] Le Nguyen D, Heitz A, Chiche L, Castro B, Boigegrain RA, et al (1990) Molecular recognition between serine proteases and new bioactive microproteins with a knotted structure. Biochimie 72: 431–435. 2124146 10.1016/0300-9084(90)90067-q

[23] Ollmann MM, Wilson BD, Yang YK, Kerns JA, Chen Y, et al (1997) Antagonism of central melanocortin receptors in vitro and in vivo by agouti-related protein. Science 278: 135–138. 9311920 10.1126/science.278.5335.135

[24] Jackson PJ, McNulty JC, Yang YK, Thompson DA, Chai B, et al (2002) Design, pharmacology, and NMR structure of a minimized cystine knot with agouti-related protein activity. Biochemistry 41: 7565–7572. 12056887 10.1021/bi012000x

[25] Craik DJ, Daly NL, Bond T, Waine C (1999) Plant cyclotides: A unique family of cyclic and knotted proteins that defines the cyclic cystine knot structural motif. J Mol Biol 294: 1327–1336. 10600388 10.1006/jmbi.1999.3383

[26] Kimura RH, Levin AM, Cochran FV, Cochran JR (2009) Engineered cystine knot peptides that bind alphav beta3, alphav beta5, and alpha5 beta1 integrins with low-nanomolar affinity. Proteins 77: 359–369. 19452550 10.1002/prot.22441 PMC5792193

[27] Silverman AP, Levin AM, Lahti JL, Cochran JR (2009) Engineered cystine-knot peptides that bind alphav beta3 integrin with antibody-like affinities. J Mol Biol 385: 1064–1075. 19038268 10.1016/j.jmb.2008.11.004 PMC2925133

[28] Jiang H, Moore SJ, Liu S, Liu H, Miao Z, et al (2012) A novel radiofluorinated agouti-related protein for tumor angiogenesis imaging. Amino Acids doi:10.1007/s00726-00012-01391-y 10.1007/s00726-012-1391-y 22945905

[29] Jiang L, Kimura RH, Miao Z, Silverman AP, Ren G, et al (2010) Evaluation of a ^64^Cu-labeled cystine-knot peptide based on agouti-related protein for PET of tumors expressing alphav beta3 integrin. J Nucl Med 51: 251–258. 20124048 10.2967/jnumed.109.069831 PMC4143171

[30] Kimura RH, Cheng Z, Gambhir SS, Cochran JR (2009) Engineered knottin peptides: a new class of agents for imaging integrin expression in living subjects. Cancer Res 69: 2435–2442. 19276378 10.1158/0008-5472.CAN-08-2495 PMC2833353

[31] Kimura RH, Miao Z, Cheng Z, Gambhir SS, Cochran JR (2010) A dual-labeled knottin peptide for PET and near-infrared fluorescence imaging of integrin expression in living subjects. Bioconjug Chem 21: 436–444. 20131753 10.1021/bc9003102 PMC3004996

[32] Liu S, Liu H, Ren G, Kimura RH, Cochran JR, et al (2012) PET imaging of integrin positive tumors using ^18^F-labeled knottin peptides. Theranostics 1: 403–412. 10.7150/thno/v01p0403 PMC3248644 22211146

[33] Miao Z, Ren G, Liu H, Kimura RH, Jiang L, et al (2009) An engineered knottin peptide labeled with ^18^F for PET imaging of integrin expression. Bioconjug Chem 20: 2342–2347. 19908826 10.1021/bc900361g PMC2804269

[34] Nielsen CH, Kimura RH, Withofs N, Tran PT, Miao Z, et al (2010) PET imaging of tumor neovascularization in a transgenic mouse model with a novel ^64^Cu-DOTA-knottin peptide. Cancer Res 70: 9022–9030. 21062977 10.1158/0008-5472.CAN-10-1338 PMC3057960

[35] Jiang L, Miao Z, Kimura RH, Silverman AP, Ren G, et al (2012) ^111^In-labeled cystine-knot peptides based on the Agouti-related protein for targeting tumor angiogenesis. J Biomed Biotechnol 2012 Article ID 368075. 10.1155/2012/368075 PMC3336217 22570527

[36] Willmann JK, Kimura RH, Deshpande N, Lutz AM, Cochran JR, et al (2010) Targeted contrast-enhanced ultrasound imaging of tumor angiogenesis with contrast microbubbles conjugated to integrin-binding knottin peptides. J Nucl Med 51: 433–440. 20150258 10.2967/jnumed.109.068007 PMC4111897

[37] Brooks PC, Clark RA, Cheresh DA (1994) Requirement of vascular integrin alpha v beta 3 for angiogenesis. Science 264: 569–571. 7512751 10.1126/science.7512751

[38] Kim S, Bell K, Mousa SA, Varner JA (2000) Regulation of angiogenesis in vivo by ligation of integrin alpha5beta1 with the central cell-binding domain of fibronectin. Am J Pathol 156: 1345–1362. 10751360 10.1016/s0002-9440(10)65005-5 PMC1876892

[39] Brooks PC, Montgomery AM, Rosenfeld M, Reisfeld RA, Hu T, et al (1994) Integrin alpha v beta 3 antagonists promote tumor regression by inducing apoptosis of angiogenic blood vessels. Cell 79: 1157–1164. 7528107 10.1016/0092-8674(94)90007-8

[40] Friedlander M, Brooks PC, Shaffer RW, Kincaid CM, Varner JA, et al (1995) Definition of two angiogenic pathways by distinct alpha v integrins. Science 270: 1500–1502. 7491498 10.1126/science.270.5241.1500

[41] Gaertner FC, Schwaiger M, Beer AJ (2010) Molecular imaging of avb3 expression in cancer patients. Q J Nucl Med Mol Imaging 20639816

[42] Cai W, Rao J, Gambhir SS, Chen X (2006) How molecular imaging is speeding up antiangiogenic drug development. Mol Cancer Ther 5: 2624–2633. 17121909 10.1158/1535-7163.MCT-06-0395

[43] Escoubas P, Rash L (2004) Tarantulas: eight-legged pharmacists and combinatorial chemists. Toxicon 43: 555–574. 15066413 10.1016/j.toxicon.2004.02.007

[44] Wood DL, Miljenovic T, Cai S, Raven RJ, Kaas Q, et al (2009) ArachnoServer: a database of protein toxins from spiders. BMC Genomics 10: 375. 19674480 10.1186/1471-2164-10-375 PMC2907703

[45] Adams ME, Mintz IM, Reily MD, Thanabal V, Bean BP (1993) Structure and properties of omega-agatoxin IVB, a new antagonist of P-type calcium channels. Mol Pharmacol 44: 681–688. 8232218

[46] Kim JI, Konishi S, Iwai H, Kohno T, Gouda H, et al (1995) Three-dimensional solution structure of the calcium channel antagonist omega-agatoxin IVA: consensus molecular folding of calcium channel blockers. J Mol Biol 250: 659–671. 7623383 10.1006/jmbi.1995.0406

[47] Rink H, Sieber P, Raschdorf F (1984) Coversion of NG-urethane protected arginine to ornithine in peptide solid phase synthesis. Tetrahedron Letters 25: 621–624.

[48] Rubina A, Bespalova Zh D, Bushuev VN (2000) The solid-phase synthesis of peptides containing an arginine residue with an unprotected guanidine group. Russian Journal of Bioorganic Chemistry 26: 235–244. 10857018

[49] Zhang Y, Skolnick J (2005) TM-align: a protein structure alignment algorithm based on the TM-score. Nucleic Acids Res 33: 2302–2309. 15849316 10.1093/nar/gki524 PMC1084323

[50] Blystone SD, Graham IL, Lindberg FP, Brown EJ (1994) Integrin alphav beta3 differentially regulates adhesive and phagocytic functions of the fibronectin receptor alpha5 beta1. J Cell Biol 127: 1129–1137. 7525603 10.1083/jcb.127.4.1129 PMC2200054

[51] Xiong JP, Stehle T, Goodman SL, Arnaout MA (2003) Integrins, cations and ligands: making the connection. J Thromb Haemost 1: 1642–1654. 12871301 10.1046/j.1538-7836.2003.00277.x

[52] Haubner R, Finsinger D, Kessler H (1997) Stereoisomeric peptide libaries and peptidomimetics for designing selective inhibitors of the avb3 integrin for a new cancer therapy. Angew Chem Int Ed 36: 1374–1389.

[53] Cochran FV, Cochran JR (2010) Phage display and molecular imaging: expanding fields of vision in living subjects. Biotechnology and Genetic Engineering Reviews 27: 57–94. 21415893 10.1080/02648725.2010.10648145

[54] McNulty JC, Jackson PJ, Thompson DA, Chai B, Gantz I, et al (2005) Structures of the agouti signaling protein. J Mol Biol 346: 1059–1070. 15701517 10.1016/j.jmb.2004.12.030

[55] Haubner R (2006) Alphavbeta3-integrin imaging: a new approach to characterise angiogenesis? Eur J Nucl Med Mol Imaging 33 Suppl 1 54–63. 16791598 10.1007/s00259-006-0136-0

[56] Beer AJ, Schwaiger M (2008) Imaging of integrin alphavbeta3 expression. Cancer Metastasis Rev 27: 631–644. 18523730 10.1007/s10555-008-9158-3

[57] Zahnd C, Kawe M, Stumpp MT, de Pasquale C, Tamaskovic R, et al (2010) Efficient tumor targeting with high-affinity designed ankyrin repeat proteins: effects of affinity and molecular size. Cancer Res 70: 1595–1605. 20124480 10.1158/0008-5472.CAN-09-2724

[58] Orlova A, Magnusson M, Eriksson TL, Nilsson M, Larsson B, et al (2006) Tumor imaging using a picomolar affinity HER2 binding affibody molecule. Cancer Res 66: 4339–4348. 16618759 10.1158/0008-5472.CAN-05-3521

[59] Schmidt MM, Wittrup KD (2009) A modeling analysis of the effects of molecular size and binding affinity on tumor targeting. Mol Cancer Ther 8: 2861–2871. 19825804 10.1158/1535-7163.MCT-09-0195 PMC4078872

[60] Wittrup KD, Thurber GM, Schmidt MM, Rhoden JJ (2012) Practical theoretic guidance for the design of tumor-targeting agents. Methods Enzymol 503: 255–268. 22230572 10.1016/B978-0-12-396962-0.00010-0 PMC3978464

[61] Hackel BJ, Kimura RH, Gambhir SS (2012) Use of ^64^Cu-labeled fibronectin domain with EGFR-overexpressing tumor xenograft: molecular imaging. Radiology 263: 179–188. 22344401 10.1148/radiol.12111504 PMC3309798

[62] Kaur S, Venktaraman G, Jain M, Senapati S, Garg PK, et al (2012) Recent trends in antibody-based oncologic imaging. Cancer Lett 315: 97–111. 22104729 10.1016/j.canlet.2011.10.017 PMC3249014

[63] Lofblom J, Feldwisch J, Tolmachev V, Carlsson J, Stahl S, et al (2010) Affibody molecules: engineered proteins for therapeutic, diagnostic and biotechnological applications. FEBS Lett 584: 2670–2680. 20388508 10.1016/j.febslet.2010.04.014

[64] Wu AM, Olafsen T (2008) Antibodies for molecular imaging of cancer. Cancer J 14: 191–197. 18536559 10.1097/PPO.0b013e31817b07ae

[65] Akizawa H, Uehara T, Arano Y (2008) Renal uptake and metabolism of radiopharmaceuticals derived from peptides and proteins. Adv Drug Deliv Rev 60: 1319–1328. 18508156 10.1016/j.addr.2008.04.005

[66] Vegt E, de Jong M, Wetzels JF, Masereeuw R, Melis M, et al (2010) Renal toxicity of radiolabeled peptides and antibody fragments: mechanisms, impact on radionuclide therapy, and strategies for prevention. J Nucl Med 51: 1049–1058. 20554737 10.2967/jnumed.110.075101

[67] Kimura RH, Teed R, Hackel BJ, Pysz MA, Chuang CZ, et al (2012) Pharmacokinetically stabilized cystine knot peptides that bind alphav beta6 integrin with single-digit nanomolar affinities for detection of pancreatic cancer. Clin Cancer Res 18: 839–849. 22173551 10.1158/1078-0432.CCR-11-1116 PMC3271184

[68] Hackel BJ, Sathirachinda A, Gambhir SS (2012) Designed hydrophilic and charge mutations of the fibronectin domain: towards tailored protein biodistribution. Protein Eng Des Sel 25: 639–648. 22691700 10.1093/protein/gzs036 PMC3449399

[69] Maillere B, Mourier G, Herve M, Cotton J, Leroy S, et al (1995) Immunogenicity of a disulphide-containing neurotoxin: presentation to T-cells requires a reduction step. Toxicon 33: 475–482. 7570632 10.1016/0041-0101(94)00186-c

[70] Zhang X, Xiong Z, Wu Y, Cai W, Tseng JR, et al (2006) Quantitative PET imaging of tumor integrin alphav beta3 expression with ^18^F-FRGD2. J Nucl Med 47: 113–121. 16391195 PMC4160026

