# Supplementary figures and images for: Engineering Agatoxin, a Cystine-Knot Peptide from Spider Venom, as a Molecular Probe for In Vivo Tumor Imaging
Source: PLoS One. 2013 Apr 3;8(4):e60498. doi: 10.1371/journal.pone.0060498 (PMC3616073; doi:10.1371/journal.pone.0060498)

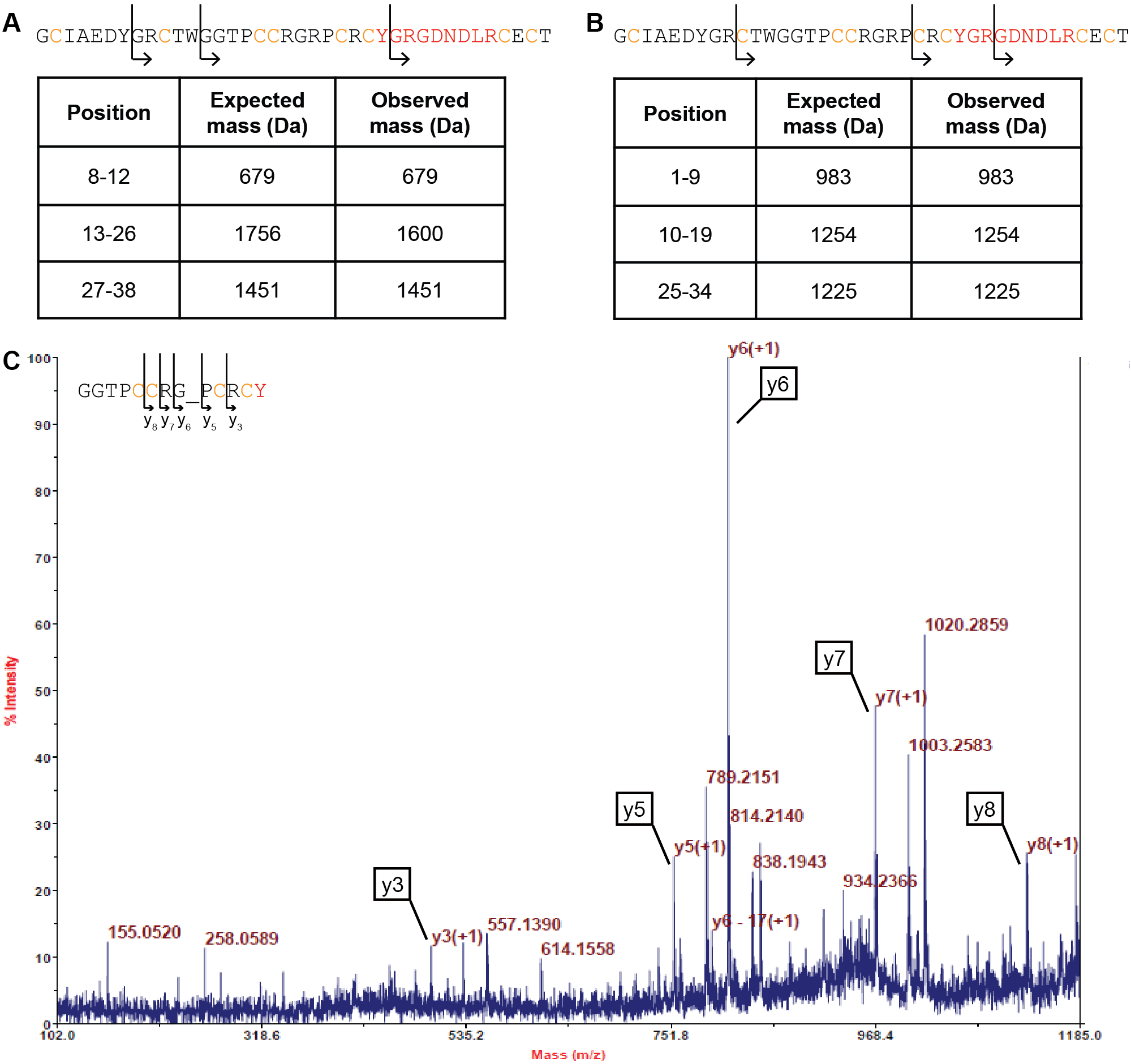

Supplement: Figure S1 — Enzymatic digestion and tandem mass spectrometry analysis of folded AgTx 7C reveals an arginine deletion at position 21. The modified AgTx 7C knottin (observed mass = 4040 Da) was reduced with dithiothreitol and alkylated with iodoacetamide. (A) Comparison of MALDI-TOF-MS of a chymotryptic digest to in silico chymotrypsin digestion using ExPASy PeptideMass revealed that the mass discrepancy is located in the sequence GGTPCCRGRPCRCY (position 13–26). (B) Comparison of tryptic digest to a Mascot search revealed that Arg19 is present and Arg24 is likely present due to the existence of fragment 25–34, indicating cleavage by trypsin after residue 24. This data suggests that the missing Arg is located at residue 21. (C) MS/MS analysis of the 1600 Da chymotryptic peptide further supports the sequence GGTPCCRG_PCRCY, with Arg21 as the most likely deletion, by the observation of y3, y5, y6, y7, and y8 ions. (TIF) [file pone.0060498.s001.tif]

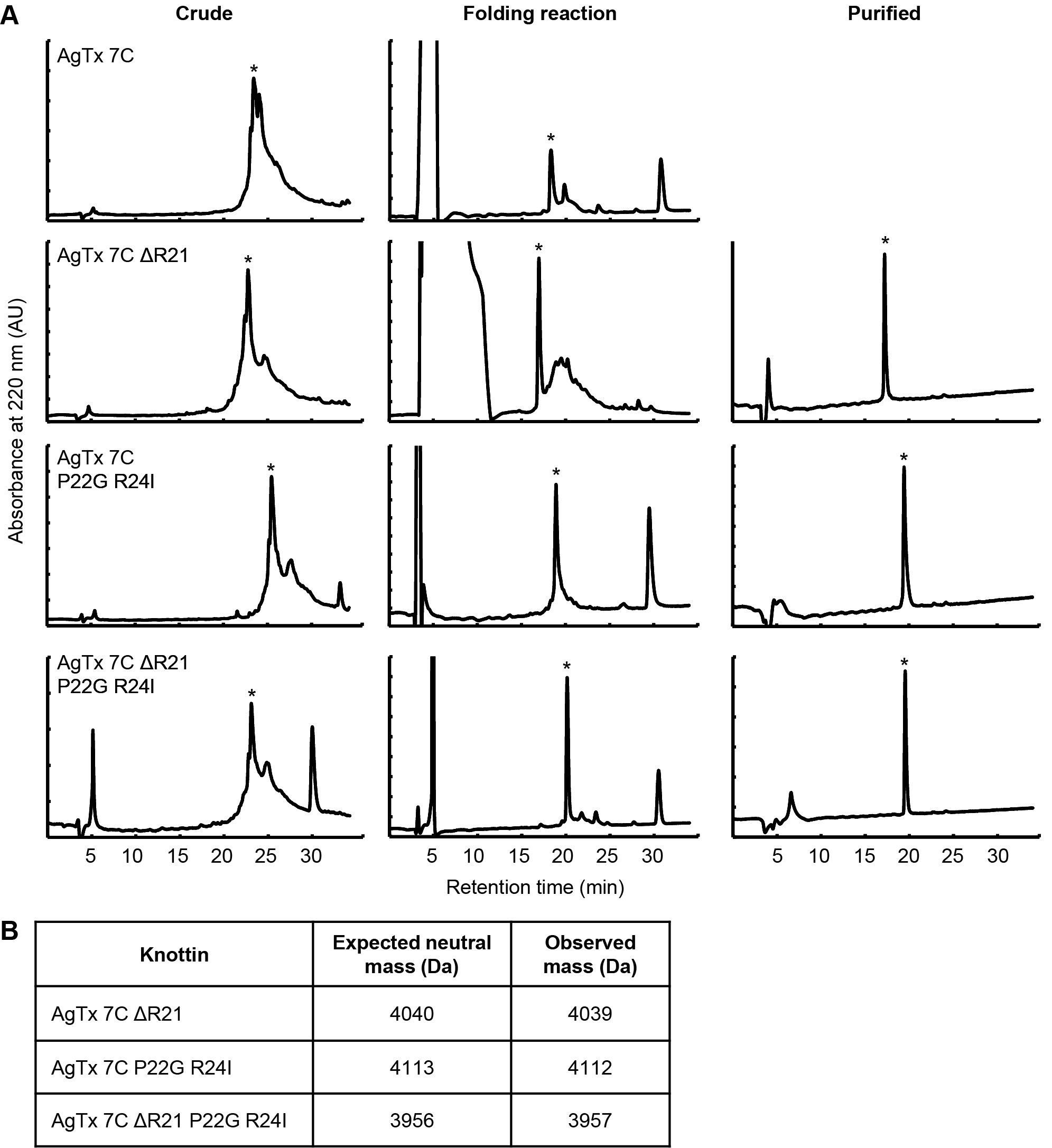

Supplement: Figure S2 — Modifications to the AgTx scaffold promote in vitro folding of integrin-binding variants. Analytical-scale RP-HPLC traces of linear, crude peptide (left), folding reaction (center), and purified, folded peptide (right) for AgTx 7C variants. Yield of purified, folded AgTx 7C was too low for further analysis. AgTx 7C P22G R24I and AgTx 7C ΔR21 P22G R24I were efficiently separated from misfolded isomers when folded from purified, linear precursor peptide, but not when folded from unpurified, crude peptide under the conditions tested. Thus, for these variants, crude linear peptide was first purified by preparatory-scale RP-HPLC using a Vydac C18 column before folding. In contrast, purification of the AgTx 7C linear precursor prior to folding still resulted in very low folding efficiency. (B) Masses of folded, purified knottins were determined by ESI-MS or MALDI-TOF-MS. (TIF) [file pone.0060498.s002.tif]

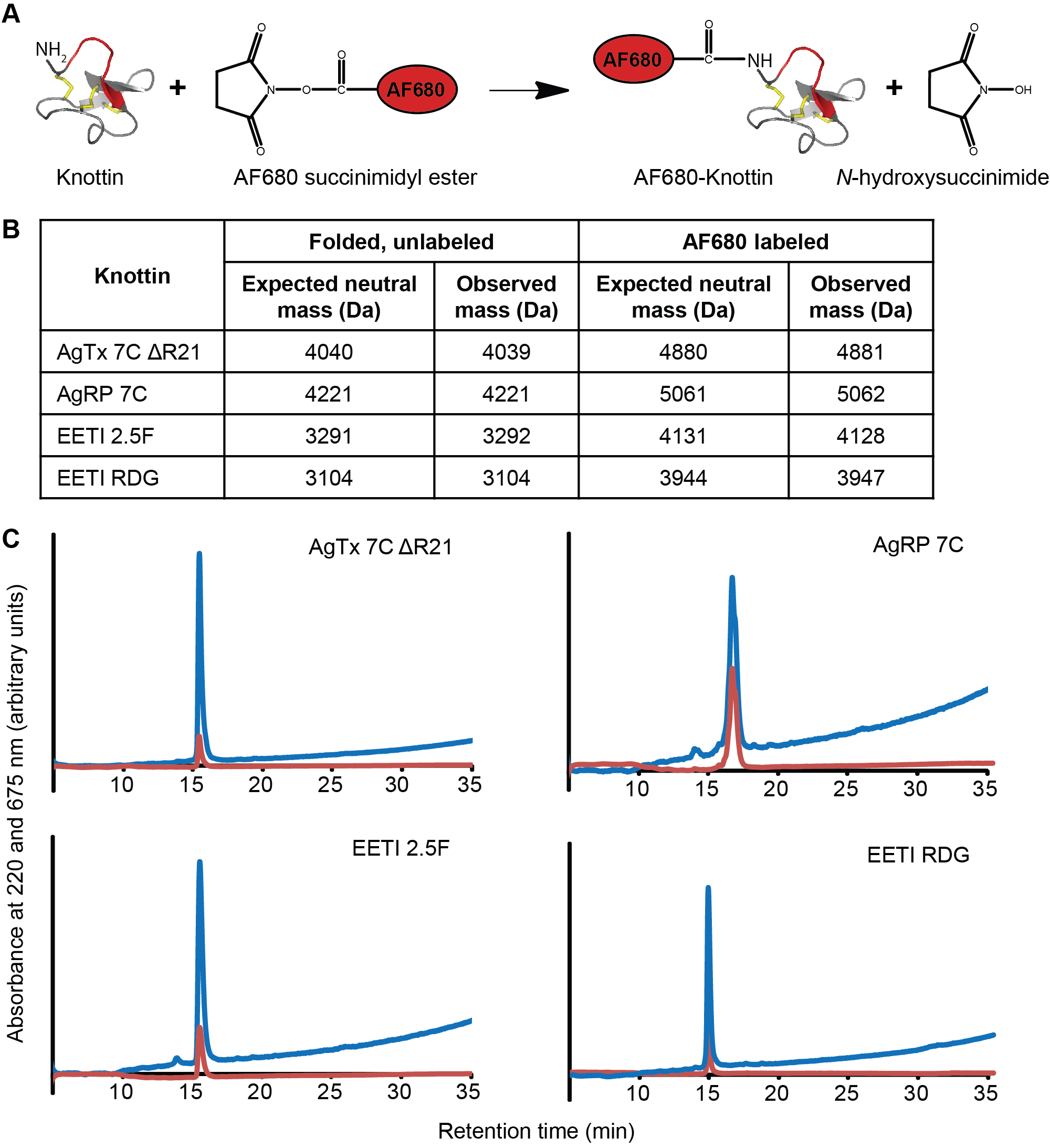

Supplement: Figure S3 — AF680 conjugation and characterization. (A) The near infrared dye AF680 was site-specifically conjugated to knottins at their N-terminal amino group using succinimidyl ester chemistry. (B) Folded, purified knottins and AF680-labeled knottins were analyzed by mass spectrometry. Expected error in these measurements is 0.1%. (C) Analysis of purified AF680-labeled knottins by analytical-scale RP-HPLC. Purity was determined to be greater than 95%. Blue traces: absorbance at 220 nm by amide bonds, red traces: absorbance at 675 nm by AF680 fluorophore. (TIF) [file pone.0060498.s003.tif]

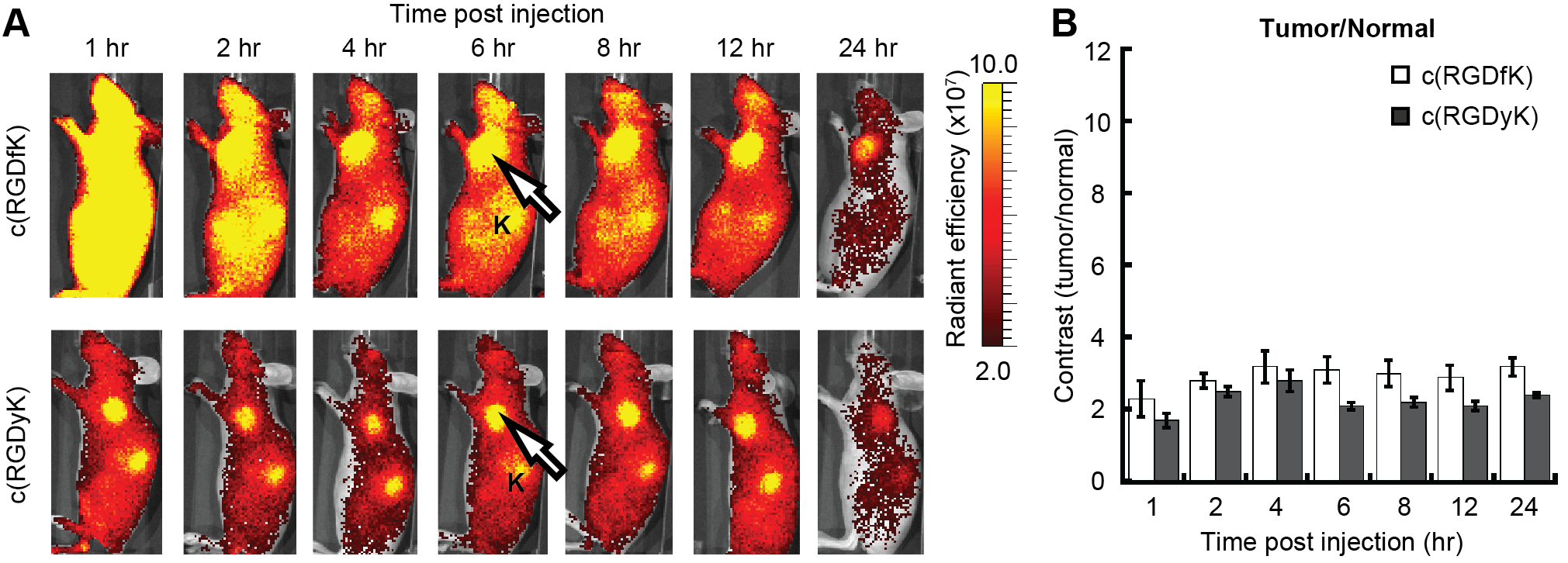

Supplement: Figure S4 — Non-invasive in vivo imaging with AF680-labeled cyclic RGD peptidomimetics. (A) Mice bearing U87MG tumor xenografts were injected with 1.5 nmol AF680-c(RGDfK) or AF680-c(RGDyK), which exhibited high tumor uptake but slow clearance from non-target tissues. Tumors (white arrow) and kidneys (K) are indicated. (B) Maximum tumor-to-normal tissue contrast ratios of 3.2±0.5 and 2.8±0.3 were measured for AF680-c(RGDfK) and AF680-c(RGDyK), respectively. Error bars represent ± SE, n = 3. (TIF) [file pone.0060498.s004.tif]
