# Supplementary material for: Engineering Agatoxin, a Cystine-Knot Peptide from Spider Venom, as a Molecular Probe for In Vivo Tumor Imaging
Source: PLoS One. 2013 Apr 3;8(4):e60498. doi: 10.1371/journal.pone.0060498 (PMC3616073; doi:10.1371/journal.pone.0060498)
Supplement: Text S1 — Supplemental materials and methods. (DOCX) [file pone.0060498.s005.docx]

**Text S1. Supplemental Materials and Methods**

Oxidative folding conditions were optimized for each knottin peptide and are described below. For AgTx P22G R24I and AgTx ΔR21 P22G R24I, linear precursor peptide was first purified on a preparatory RP-HPLC C18 column before folding. For AgTx 7C ΔR21, AgRP 7C, EETI 2.5F, and EETI RDG, folding was performed on the crude synthetic peptide, without intermediate purification of the linear precursor. For AgTx 7C, folding was attempted with both crude and purified linear precursors under the conditions described below, but resulted in inefficient folding.

Folding from crude linear precursor:

AgTx 7C, AgTx 7C ΔR21:

Folding buffer contained 10 mM reduced glutathione, 1 mM oxidized glutathione, 10% (v/v) DMSO, 4 M guanidine-HCl, in 0.1 M ammonium bicarbonate, pH 8.0 at 4°C with gentle rocking for 3 days.

AgRP 7C:

Folding buffer contained 10 mM reduced glutathione, 1 mM oxidized glutathione, 10% (v/v) DMSO, 4 M guanidine-HCl, in 0.1 M ammonium bicarbonate, pH 8.0 at room temperature with gentle rocking overnight to two days.

EETI 2.5F, EETI RDG:

Folding buffer contained 2.5 mM reduced glutathione and 20% (v/v) dimethylsulfoxide (DMSO) in 0.1 M ammonium bicarbonate, pH 8.0 at room temperature with gentle rocking overnight.

Folding from purified linear precursor:

AgTx 7C, AgTx P22G R24I, AgTx ΔR21 P22G R24I:

Folding buffer contained 10 mM reduced glutathione and 1 mM oxidized glutathione in 0.1 M Tris-HCl, pH 8.0 at 4°C with gentle rocking for 24 hours.
